# Surgical Techniques and Related Perioperative Outcomes After Robot-assisted Minimally Invasive Gastrectomy (RAMIG): Results From the Prospective Multicenter International Ugira Gastric Registry

**DOI:** 10.1097/SLA.0000000000006147

**Published:** 2023-11-03

**Authors:** Cas de Jongh, Fabio Cianchi, Takahiro Kinoshita, Feike Kingma, Micaela Piccoli, Attila Dubecz, Ewout Kouwenhoven, Marc van Det, Tom Mala, Andrea Coratti, Paolo Ubiali, Paul Turner, Pursnani Kish, Felice Borghi, Arul Immanuel, Magnus Nilsson, Ioannis Rouvelas, Jens P. Hӧlzen, Philippe Rouanet, Olivier Saint-Marc, David Dussart, Alberto Patriti, Francesca Bazzocchi, Boudewijn van Etten, Jan W. Haveman, Marco DePrizio, Flávio Sabino, Massimo Viola, Felix Berlth, Peter P. Grimminger, Franco Roviello, Richard van Hillegersberg, Jelle Ruurda, UGIRA Collaborative Group

**Affiliations:** * Department of Surgery, University Medical Center (UMC) Utrecht, University of Utrecht, Utrecht, The Netherlands; † Department of Experimental and Clinical Medicine, University Hospital Careggi, University of Florence, Florence, Italy; ‡ Department of Gastric Surgery, National Cancer Center Hospital East, Kashiwa, Japan; § Department of Surgery, Civile Baggiovara Hospital, Azienda Ospedaliero-Universitaria (AOU) of Modena, Modena, Italy; ∥ Department of Surgery, Klinikum Nürnberg, Paracelsus Medical University, Nürnberg, Germany; ¶ Department of Surgery, Hospital ZGT Almelo, Almelo, The Netherlands; # Department of Surgery, Oslo University Hospital, University of Oslo, Norway; ** Department of Surgery, Misericordia Hospital Grosseto, Grosseto, Italy; †† Department of Surgery, Hospital Santa Maria degli Angeli, Pordenone, Italy; ‡‡ Department of Surgery, Lancashire Teaching Hospitals NHS Foundation Trust, Preston, UK; §§ Department of Surgery, General Hospital Cuneo, Cuneo, Italy; ∥∥ Department of Surgery, Candiolo Cancer Institute, Turin, Italy; ¶¶ Department of Surgery, Newcastle upon Tyne Hospitals NHS Foundation Trust, Newcastle, UK; ## Department of Upper Abdominal Diseases, Division of Surgery and Oncology, CLINTEC, Karolinska Institutet and Karolinska University Hospital, Stockholm, Sweden; *** Department of Surgery, UMC Münster, Münster, Germany; ††† Department of Surgery, Montpellier Cancer Institute, Montpellier, France; ‡‡‡ Department of Surgery, Centre Hospitalier Régional Universitaire Orléans, Orléans, France; §§§ Department of Surgery, General Hospital Marche Nord, Pesaro, Italy; ∥∥∥ Department of Surgery, San Giovanni Rotondo Hospital IRCCS, San Giovanni Rotondo, Italy; ¶¶¶ Department of Surgery, UMC Groningen, University of Groningen, The Netherlands; ### Department of Surgery, General Hospital Arezzo, Arezzo, Italy; **** Department of Surgery, National Cancer Institute Rio de Janeiro, Rio de Janeiro, Brasil; †††† Department of Surgery, General Hospital Tricase, Tricase, Italy; ‡‡‡‡ Department of Surgery, UMC Mainz, Mainz, Germany; §§§§ Department of Surgery, University Hospital Siena, Siena, Italy

**Keywords:** gastric cancer, minimally invasive gastrectomy, robot-assisted gastrectomy, standardization

## Abstract

**Objective:**

To gain insight into the global practice of robot-assisted minimally invasive gastrectomy (RAMIG) and evaluate perioperative outcomes using an international registry.

**Background:**

The techniques and perioperative outcomes of RAMIG for gastric cancer vary substantially in the literature.

**Methods:**

Prospectively registered RAMIG cases for gastric cancer (≥10 per center) were extracted from 25 centers in Europe, Asia, and South-America. Techniques for resection, reconstruction, anastomosis, and lymphadenectomy were analyzed and related to perioperative surgical and oncological outcomes. Complications were uniformly defined by the Gastrectomy Complications Consensus Group.

**Results:**

Between 2020 and 2023, 759 patients underwent total (n=272), distal (n=465), or proximal (n=22) gastrectomy (RAMIG). After total gastrectomy with Roux-en-Y-reconstruction, anastomotic leakage rates were 8% with hand-sewn (n=9/111) and 6% with linear stapled anastomoses (n=6/100). After distal gastrectomy with Roux-en-Y (67%) or Billroth-II-reconstruction (31%), anastomotic leakage rates were 3% with linear stapled (n=11/433) and 0% with hand-sewn anastomoses (n=0/26). Extent of lymphadenectomy consisted of D1+ (28%), D2 (59%), or D2+ (12%). Median nodal harvest yielded 31 nodes (interquartile range: 21–47) after total and 34 nodes (interquartile range: 24–47) after distal gastrectomy. R0 resection rates were 93% after total and 96% distal gastrectomy. The hospital stay was 9 days after total and distal gastrectomy, and was median 3 days shorter without perianastomotic drains versus routine drain placement. Postoperative 30-day mortality was 1%.

**Conclusions:**

This large multicenter study provided a worldwide overview of current RAMIG techniques and their respective perioperative outcomes. These outcomes demonstrated high surgical quality, set a quality standard for RAMIG, and can be considered an international reference for surgical standardization.

Gastric cancer ranks third in global cancer mortality.^1^ Locally advanced cancer is treated by D2-gastrectomy with curative intent, mostly combined with perioperative or adjuvant chemotherapy.^2–5^ Although a traditional open approach for gastrectomy provides good oncological results, minimally invasive gastrectomy (MIG) has been increasingly implemented over recent years.^6,7^

Randomized-controlled trials comparing open versus conventional MIG showed similar oncological results in terms of lymph node yield, R0-resections and survival.^8–13^ Whereas Western studies found similar morbidity, Asian trials showed lower morbidity, faster postoperative recovery and better quality of life after MIG.^8–13^ Although these findings are promising, conventional MIG is a complex procedure associated with a substantial learning curve.^14–16^ Furthermore, laparoscopic surgery involves technical limitations, such as impaired depth perception, limited range-of-motion and an ergonomically suboptimal posture when operating, leading to musculoskeletal disorders.^17,18^ Robot-assisted minimally invasive gastrectomy RAMIG) could overcome these challenges with 3-dimensional magnified visualization, a stable optical platform controlled by the primary operating surgeon, tremor suppression, and hand-wristed articulation of robotic instruments.^18^ These advantages improve dexterity, optimize surgical precision, and facilitate complex manoeuvres including anastomotic techniques, lymphadenectomy, and suturing. In addition, the RAMIG learning curve may be relatively short, especially for surgeons experienced in MIG.^19–23^

Current evidence on the safety, feasibility, and efficacy of RAMIG consists of single-center case-series, some multicenter studies and 4 randomized trials.^18,23–32^ Between studies, RAMIG surgical techniques and perioperative outcomes appear to vary substantially. Furthermore, different definitions of complications were utilized complicating comparison across studies.^33–35^ The Upper-GI International Robotic Association (UGIRA) established an international registry to gain insight into global practices and ultimately determine the optimal surgical gastric cancer treatment.^36^ Using the registry, this study inventoried current RAMIG techniques and evaluated their respective perioperative outcomes with uniform definitions.

## METHODS

### Upper-GI International Robotic Association

Since the founding of UGIRA in 2017, UGIRA aims to guide the implementation of robotic techniques in upper-gastrointestinal surgery by effective training pathways, perform international research, and establish standardized procedure guidelines. The establishment of the UGIRA Esophageal Registry in 2018 motivated an increasing number of robotic upper-gastrointestinal surgeons to join UGIRA, resulting in several scientific papers using the registry.^37,38^ After establishing the UGIRA Gastric Registry in 2020, prospective RAMIG cases were registered until the present day. The current study is the first research based on the UGIRA Gastric Registry.

### Design

All RAMIG cases with histologic confirmation of resectable gastric cancer were included until February 2023. Centers with <10 cases were considered not eligible for participation and were excluded. Other exclusion criteria consisted of squamous cell carcinoma, benign indications or other histology (eg, gastrointestinal stromal tumors or neuroendocrine differentiation), wedge resections or (palliative) surgery without surgical resection of the primary tumor, and previous gastric surgery. In total, 25 centers from Europe, Asia, and South-America participated in this study, as listed in Supplementary Methods, Supplemental Digital Content 1, http://links.lww.com/SLA/E936 and Supplementary Figure 1, Supplemental Digital Content 1, http://links.lww.com/SLA/E936. Participating surgeons were considered proficient in open and minimally invasive gastrectomy and had surgical experience varying between 10 and 110 RAMIG cases. Central ethics approval was obtained in UMC Utrecht, waiving informed consent (20/134), and institutional review board approval was acquired in each participating center. All procedures followed were in accordance with the ethical standards of the responsible committee on human experimentation (institutional and national) and with the Helsinki Declaration of 1964 and later versions.

### Prospective Data Collection

The proposed items for the data collection were determined in a consensus meeting by the UGIRA Collaborative Group. All data were collected prospectively. RAMIG cases were registered consecutively and in chronological order. The registry was hosted by Castor EDC, a secure online data-capturing platform that meets international privacy, ethical, and regulatory requirements.^39^ Baseline data consisted of patient demographics including age, sex, body mass index (BMI), weight loss, American Society of Anesthesiologists (ASA) classification, comorbidities, previous surgery, disease stage according to the 8th edition of TNM staging by the American Joint Committee on Cancer, and neoadjuvant therapy.^40^ Intraoperative data consisted of operating time, blood loss, conversion, complications, and RAMIG techniques for the surgical resection, reconstruction, anastomosis, and lymphadenectomy. Histopathologic data consisted of tumor histology, lymph node yield, and resection margin status. Nodal stations were based on the 5th guidelines of the Japanese Gastric Cancer Association (JGCA).^41^ Complications were uniformly defined according to the Gastrectomy Complications Consensus Group (GCCG) and graded using the Clavien-Dindo classification.^34,42^ For postoperative recovery, hospital and intensive care unit stay, reoperations, application of Enhanced Recovery After Surgery (ERAS) guidelines, re-admission within 30 days after discharge, and 30-day mortality were recorded.^43^

No identifiable patient data were registered to safeguard patient privacy. Therefore, cases were registered at once after the 30-day follow-up period. To ensure data quality and minimize registration error, automated built-in data verification steps were implemented; missing items were highlighted in color automatically, and an audit trail registered all adjustments. The registry coordinator (C.d.J.) instructed centers individually for the data entry and performed additional data cleaning to verify registered data and check the completeness of data entry.

### Outcomes

The main outcomes included techniques used for resection, reconstruction, anastomosis, and lymphadenectomy. These technical factors were analyzed and related to perioperative surgical and oncological outcomes. Furthermore, textbook outcome was assessed, which was defined as a composite measure including R0 resection, nodal yield ≥15 nodes, no intraoperative complications, no severe postoperative complications (≥3b Clavien-Dindo grading), no reoperations, no ICU admission, hospitalization <21 days, and no 30-day mortality.

### Statistical Analysis

Patients were categorized according to the extent of gastrectomy (total, distal, or proximal gastrectomy) and outcomes were descriptively reported for these 3 subgroups. Depending on data distribution, continuous variables were presented as means with SD or medians with range or interquartile range (IQR). Categorical variables were displayed as frequencies with percentages (%). Analyses were performed using IBM SPSS Statistics version 27.0 (SPSS Inc., Chicago, IL).

## RESULTS

**FIGURE 1:**
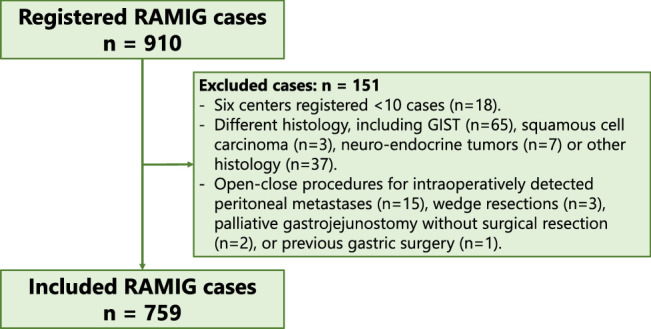
Study flow chart.

Between June 2020 and February 2023, 759 of 910 registered patients were included (Fig. 1). Reasons for exclusion (n=151) were other histology (n=112), centers with <10 registered RAMIG cases (n=18; 6 centers), no surgical resection due to intraoperatively detected peritoneal carcinomatosis (n=15), palliative gastrojejunostomy (n=2), wedge resections (n=3), or previous gastric surgery (n=1).

Baseline characteristics are displayed in Table 1 (n=759). Patients had a median age of 70 years (range: 19–93) and were mostly male (n=425; 56%). Mean BMI was 24.8 kg/m^2^ (SD: ±4.4). Preoperative weight loss was frequently observed (n=257; 47%). Most patients showed ASA classification 2 (n=438; 59%) or 3 (n=233; 32%). Tumors were localized in the gastric cardia (11%), fundus/corpus (37%), antrum/pylorus (48%), or diffusely located throughout the stomach (4%). Most patients underwent upfront surgical resection (55%) or neoadjuvant chemotherapy (42%), whereas other neoadjuvant treatment (3%) was administered infrequently. Western patients had higher age (median: 70 vs. 69 years), BMI (mean 25.2 vs. 22.8 kg/m^2^), ASA classification (ASA-3: 36% vs. 2%) and comorbidities (69% vs. 57%) than Eastern patients (Supplementary Table 1, Supplemental Digital Content 1, http://links.lww.com/SLA/E936).

**TABLE 1:** Characteristics of all Patients Undergoing RAMIG (n=759)

Characteristics	Entire cohort, n=759 (100%)	Missing values
Age, years [median (range)]	70 (19–93)	0
Sex		0
Male	425 (56)	
Female	334 (44)	
BMI, kg/m^2^ [mean (SD))	24.8±4.4	107 (14)
Weight loss		206 (27)
No	295 (53)	
Yes	258 (47)	
ASA classification		25 (3)
1	56 (8)	
2	438 (59)	
3	233 (32)	
4	7 (1)	
Previous thoracic or intra-abdominal surgery (yes)	229 (31)	17 (2)
Any comorbidity	497 (68)	23 (3)
Pulmonary comorbidity	89 (12)	23 (3)
Cardiovascular comorbidity	344 (47)	23 (3)
Gastrointestinal comorbidity	65 (9)	23 (3)
Histology		0
Adenocarcinoma	755 (99.5)	
Adenosquamous cell carcinoma	4 (0.5)	
Tumor location		9 (1)
Cardia/esophagogastric junction	83 (11)	
Fundus/corpus	275 (37)	
Antrum/pylorus	361 (48)	
Diffuse through the stomach	31 (4)	
Lauren classification		136 (18)
Intestinal type*	401 (64)	
Diffuse type	222 (36)	
Differentiation grade		78 (10)
Good—moderate differentiation	307 (45)	
Poor—undifferentiated	374 (55)	
Clinical T stage		43 (6)
cT1	118 (17)	
cT2	178 (25)	
cT3	271 (38)	
cT4a	90 (13)	
cT4b	10 (1)	
cTx	49 (7)	
Clinical N stage		42 (6)
cN0	369 (51)	
cN+ (cN1–cN3)	313 (44)	
cNx	35 (5)	
Clinical M stage		37 (5)
cM0	674 (93)	
cM1	19 (3)	
cMx	29 (4)	
Neoadjuvant therapy		11 (2)
None	409 (55)	
Chemotherapy†	314 (42)	
Chemoradiotherapy‡	13 (2)	
Other	12 (2)	

Percentages may differ from 100% due to rounding.

* Mixed type tumors (n=64/623; 10%) were categorized among the intestinal type (n=401 in total combined).

† Chemotherapy consisted mostly of the FLOT regimen (n=254; 81%), triplet ECX/EOX regimen (n=12; 4%), or other regimens (n=48; 15%).

‡ Chemoradiotherapy consisted of the CROSS regimen (n=4; 31%) or other regimens (n=9; 69%).

ASA indicates American Society of Anesthesiologists; BMI, body mass index (kg/m^2^); IQR, interquartile range.

RAMIG techniques and intraoperative details are depicted in Table 2. The robotic Da Vinci Xi-system was predominantly used for RAMIG (Xi-system 87%; Si-system 10%; X-system 3%), in almost all cases (99%) using the fourth robotic arm. In total, 759 gastric cancer patients from 25 hospitals located in Europe (n=650), Asia (n=98), and South-America (n=11) underwent total (n=272; 36%), distal (n=465; 61%), or proximal (n=22; 3%) gastrectomy (RAMIG). The RAMIG techniques for surgical resection across continents in our cohort are displayed in Supplementary Figure 2, Supplemental Digital Content 1, http://links.lww.com/SLA/E936 showing the rates in Europe and Asia of total (62% and 59%), distal (37% and 27%), and proximal gastrectomies (1% and 14%).

**TABLE 2:** Surgical Techniques and Intraoperative Details for all RAMIG Procedures (n=759)

Characteristics	All patients. n=759 (100%)	Total gastrectomy, n=272 (100%)	Distal gastrectomy, n=465 (100%)	Proximal gastrectomy, n=22 (100%)	Missing values
Continent					0
Europe	650 (86)	240 (88)	402 (86)	8 (36)	
Asia	98 (13)	26 (10)	58 (13)	14 (64)	
South-America	11 (1)	6 (2)	5 (1)	0	
Robotic system					0
Da Vinci Xi	661 (87)	236 (87)	403 (87)	22 (100)	
Da Vinci Si	77 (10)	20 (7)	57 (12)	0	
Da Vinci X	21 (3)	16 (6)	5 (1)	0	
Using a fourth robotic arm					65 (9)
Yes	685 (99)	254 (99)	410 (99)	21 (96)	
No	9 (1)	3 (1)	5 (1)	1 (4)	
Type of reconstruction					5 (1)
Roux-en-Y	581 (77)	268 (100)	312 (67)	1 (5)	
Bilroth-II	145 (19)	0	144 (31)	1 (5)	
Other	28 (4)	0	8 (2)	20 (91)	
Anastomotic technique					6 (1)
Linear stapled	547 (73)	100 (37)	436 (94)	16 (73)	
Circular stapled	63 (8)	58 (22)	0	0	
Hand-sewn	143 (19)	111 (41)	26 (6)	6 (27)	
Anastomotic type					6 (1)
End-to-side	306 (41)	168 (62)	129 (28)	9 (41)	
Side-to-side	436 (58)	101 (38)	323 (70)	12 (55)	
End-to-end	11 (1)	0	10 (2)	1 (5)	
Anastomotic localization					55 (7)
Antecolic	553 (79)	169 (65)	380 (88)	4 (33)	
Retrocolic	151 (21)	92 (35)	51 (12)	8 (67)	
Anastomotic surgical approach					20 (3)
Robot-assisted	619 (84)	213 (80)	387 (86)	19 (86)	
Nonrobot-assisted	120 (16)	52 (20)	65 (14)	3 (14)	
Extent of lymphadenectomy					3 (0.4)
D1	10 (1)	2 (1)	8 (2)	0	
D1+	214 (28)	52 (19)	147 (32)	15 (68)	
D2	443 (59)	175 (64)	263 (57)	5 (23)	
D2+	89 (12)	43 (16)	44 (10)	2 (9)	
Intraoperative frozen section					0
Yes	198 (26)	91 (34)	97 (21)	12 (55)	
No	561 (74)	181 (66)	368 (79)	10 (45)	
Omentectomy					42 (6)
Total	321 (45)	160 (60)	158 (37)	3 (14)	
Partial	200 (28)	55 (21)	143 (33)	2 (9)	
No omentectomy	196 (27)	51 (19)	128 (30)	17 (77)	
Jejunal pouch reconstruction					8 (1)
Yes	5 (1)	5 (2)	0	0	
No	746 (99)	261 (98)	463 (100)	22 (100)	
Jejunal feeding tube					6 (1)
Yes	23 (3)	18 (7)	4 (1)	1 (5)	
No	730 (97)	254 (93)	459 (99)	21 (95)	
Routine drain placement					1 (0.1)
No	104 (14)	55 (20)	48 (10)	1 (5)	
Yes, 1 drain	494 (65)	169 (62)	314 (68)	11 (50)	
Yes, 2 or more drains	160 (21)	48 (18)	102 (22)	10 (45)	

Definition of the D-levels for lymphadenectomy were based on the 5th edition of the Japanese Gastric Cancer Association (JGCA), and consisted for D1 of stations 1 to 7, for D1+ stations 8, 9 and 11p were added to D1, for D2 stations 11d and 12a were added to D1+, and D2+ consisted of lymphadenectomy beyond D2 (stations 10 or 13–16).

Percentages may not add up to 100% due to rounding.

Perioperative outcomes and histopathologic results after RAMIG are listed in Table 3. Conversion to open surgery occurred during 7% of total and 4% of distal gastrectomies due to bleeding (n=7; 1%), inability to proceed due to unclear surgical plane (n=11; 1%), severe adhesions (n=4; 1%), or other (n=20; 3%).

**TABLE 3:** Perioperative Surgical Outcomes and Histopathologic Results After RAMIG (n=759)

Entire cohort: n=759				
Perioperative outcomes	Total gastrectomy, n=272 (100%)	Distal gastrectomy, n=465 (100%)	Proximal gastrectomy, n=22 (100%)	Missing values
Operating time, min, median [IQR]	331 [275–390]	270 [221–330]	360 [314–428]	29 (4)
Intraoperative blood loss, mL, median [IQR]	120 [50–200]	100 [50–200]	38 [20–67]	161 (21)
Textbook outcome*	173 (64)	338 (74)	14 (64)	6 (1)
Intraoperative complications				0
Any	27 (10)	27 (6)	0	
Conversion	18 (7)	17 (4)	0	
Bleeding	5 (2)	5 (1)	0	
Pancreatic injury	0	0	0	
Splenic injury	1 (0.4)	3 (0.6)	0	
Postoperative complications†				5 (1)
Any complication	114 (42)	108 (23)	8 (36)	
Anastomotic leakage	27 (10)	11 (2)	4 (18)	
Duodenal stump leakage	5 (2)	4 (1)	0	
Pulmonary (including pneumonia)‡	47 (17)	23 (5)	5 (23)	
Cardiac (including atrial fibrillation)§	14 (5)	12 (3)	0	
Ileus	18 (7)	12 (3)	0	
Intra-abdominal abscess	11 (4)	7 (2)	1 (5)	
Wound complication	5 (2)	4 (1)	0	
Pancreatitis or pancreatic leakage/fistula	2 (1)	8 (2)	0	
Chyle leakage	2 (1)	2 (0.4)	0	
Postoperative bleeding requiring treatment	2 (1)	12 (3)	1 (5)	
Clavien-Dindo grading (most severe complication)				3 (0.4)
Grade 0 (no complications)	157 (58)	356 (77)	14 (64)	
Grade 1	6 (2)	10 (2)	0	
Grade 2	59 (22)	45 (10)	4 (18)	
Grade 3A	22 (8)	16 (4)	3 (14)	
Grade 3B	17 (6)	23 (5)	0	
Grade 4A	7 (3)	6 (1)	1 (5)	
Grade 4B	3 (1)	1 (0.2)	0	
Grade 5 (complication resulting in death)	1 (0.4)	3 (0.6)	0	
Radicality; resection margin status∥				14 (2)
R0	251 (93)	437 (96)	20 (91)	
R1	19 (7)	16 (4)	2 (9)	
Lymph node yield, nodes, median [IQR]	31 [21–47]	34 [24–47]	34 [29–41]	20 (3)
Nodal yield: 15 lymph nodes or more	245 (92)	430 (96)	22 (100)	22 (3)
Length of hospital stay, days, median [IQR]	9 [7–14]	9 [7–11]	12 [8–21]	5 (1)
Length of ICU admission, days, median [IQR]	1 [0–2]	0 [0–1]	1 [1–2]	8 (1)
ERAS protocol applied for recovery	209 (84)	250 (61)	16 (84)	80 (11)
Re-admissions within 30 days after discharge	33 (12)	29 (6)	2 (9)	28 (4)
Postoperative mortality at 30 days	2 (1)	6 (1)	0	31 (4)

Percentages may count ±100% due to rounding.

* Textbook outcome was defined as a radical resection (R0), nodal yield ≥15 lymph nodes, no intraoperative complications, no postoperative complications ≥3b Clavien-Dindo grading, no reoperations, no ICU admission, hospital stay <21 days, and no 30-day mortality.

† Postoperative complications were classified according to the definitions from the Gastrectomy Complications Consensus Group (GCCG).

‡ Pneumonia occurred in 27 (10%), 10 (2%), and 2 (9%) patients after total, distal, and proximal gastrectomy.

§ Atrial fibrillation occurred in 12 (4%), 10 (2%), and 0 (0%) of patients after total, distal, and proximal gastrectomy.

∥ Regarding all R1 resections (n=37), the Lauren histologic subtypes were subdivided in diffuse type (n=19; 63%) or intestinal/mixed type (n=11; 37%). The remaining 7 patients (19%) had unknown Lauren subtype and were regarded as missing for the histologic subtype.

### Total Gastrectomy (RAMIG)

Total gastrectomy (n=272) was combined with Roux-en-Y (100%) reconstruction using a hand-sewn (41%), linear (37%), or circular stapled (22%) esophagojejunal anastomosis. Anastomotic leakage rates were 21% with circular stapled (n=12/57), 8% with hand-sewn (n=9/111) and 6% with linear stapled anastomoses (n=6/100; Table 4). For the Western and Eastern subcohorts (Supplementary Fig. 2, Supplemental Digital Content 1, http://links.lww.com/SLA/E936), leakage rates were 11% and 0% (n=0/26). Duodenal stump leakage was observed for 0% after hand-sewn (n=0/111), 3% after linear (n=3/100), and 4% after circular stapled (n=2/57) gastric anastomoses. For total gastrectomy, the median case volume per center were 7 (range: 1–26) for linear stapling (10 centers), 6 (range: 1–38) for hand-sewn (12 centers) and 5 (range: 1–14) for circular stapling (11 centers). Total omentectomy was often performed (60%), followed by partial (21%) or no omentectomy (19%). A jejunal pouch was occasionally created (2%) and jejunal feeding tubes were infrequently placed (7%).

**TABLE 4:** Anastomotic Leakage Rates According to Different Anastomotic Techniques After RAMIG

Entire cohort (n=748)*	Anastomotic leakage, n (%)	Duodenal stump leakage, n (%)
Total gastrectomy (n=268)		
Linear stapled anastomosis (n=100; 37%)	6 (6)	3 (3)
Circular stapled anastomosis (n=57; 21%)	12 (21)	2 (4)
Hand-sewn anastomosis (n=111; 41%)	9 (8)	0
Distal gastrectomy (n=458)		
Linear stapled anastomosis (n=433; 95%)	11 (3)	3 (1)
Circular stapled anastomosis (n=0; 0%)	–	–
Hand-sewn anastomosis (n=26; 5%)	0	1 (4)
Proximal gastrectomy (n=22)		
Linear stapled anastomosis (n=16; 73%)	4 (25)	0
Circular stapled anastomosis (n=0; 0%)	–	–
Hand-sewn anastomosis (n=6; 27%)	0	0

* There were 11 missing (1%) for anastomotic technique or leakage.

### Distal Gastrectomy (RAMIG)

During distal gastrectomy (n=465), Roux-en-Y (n=312; 67%), Billroth-II (n=144; 31%), or other (n=8; 2%) reconstructions were performed, creating the anastomosis predominantly using linear stapling (94%), or hand-sewn (6%). Anastomotic leakage rates were 3% with linear stapled (n=11/433) and 0% with hand-sewn anastomoses (n=0/26; Table 4). For the Western and Eastern subcohorts, leakage rates were 3% and 0% (n=0/58). Duodenal stump leakage was observed for 1% after linear stapled (n=3/433) and 4% after hand-sewn (n=1/26) gastric anastomoses. Total (37%), partial (33%) or no omentectomy (30%) were performed in similar proportions.

### Lymphadenectomy

Extent of lymphadenectomy (n=756) showed that ≥D1+ lymphadenectomy was performed for 99% of RAMIG cases (Table 5), consisting of D1 (1%), D1+ (28%), D2 (59%) and D2+ (12%). This is reflected in the median lymph node yield after RAMIG of 34 nodes (IQR: 24–47) in the overall cohort, and 31 nodes (IQR: 21–47) after total gastrectomy, 34 nodes (IQR: 24–47) after distal gastrectomy and 34 nodes (IQR: 29–41) after proximal gastrectomy. Intraoperative bleeding (2%), splenic (0.6%), or pancreatic injury (0%) occurred sporadically during robot-assisted D2/D2+ lymphadenectomy (n=532; Supplementary Table 2, Supplemental Digital Content 1, http://links.lww.com/SLA/E936).

**TABLE 5:** Overview of the Lymphadenectomy Types During RAMIG, Stratified Per Clinical Disease Stage

Clinical disease stage, n=756, RAMIG patients*	cT1N0 stage†, n=104 (100%)	cT1N+ or cT2-4 stage†, n=556 (100%)
Extent of lymphadenectomy‡		
All RAMIG patients (n=756)		
D1	0	5 (1)
D1+	56 (54)	125 (22)
D2	38 (37)	358 (65)
D2+	10 (10)	68 (12)
Extent of lymphadenectomy‡		
Only total gastrectomy patients (n=272)		
D1	0	1 (0.4)
D1+	8 (38)	40 (17)
D2	8 (38)	156 (67)
D2+	5 (24)	37 (16)
Extent of lymphadenectomy‡		
Only distal gastrectomy patients (n=462)		
D1	0	4 (1)
D1+	39 (53)	79 (26)
D2	29 (40)	198 (63)
D2+	5 (7)	29 (9)
Extent of lymphadenectomy‡		
Only proximal gastrectomy patients (n=22)		
D1	0	0
D1+	9 (90)	6 (50)
D2	1 (10)	4 (33)
D2+	0	2 (17)

Percentages may not add up to 100% due to rounding.

* There were 3 missing (0.4%) for extent of lymphadenectomy.

† Clinical disease stage was insufficient to be stratified in the groups (cTxN0 or cNx) for 54 patients (8%), and there were 42 missing (6%).

‡ According to the 5the definitions of the Japanese Gastric Cancer Association (JGCA) classification.

For cT1N0-stage gastric cancer (n=104), D1+ was performed most frequently (54%), followed by D2 (37%) or D2+ (10%). For cT1N+ or cT2-4-stage disease (n=556), D2 was performed most often (65%), followed by D1+ (22%) or D2+ (12%).

### Radicality

R0 resection rates were 93% after total, 96% after distal, and 91% after proximal gastrectomy. For the majority of RAMIG procedures (74%), intraoperative frozen sections were not utilized. For distal gastrectomy, refraining from intraoperative frozen sections showed 4% R1 resections, whereas 3% R1 resections were found when performing frozen sections (Supplementary Table 3, Supplemental Digital Content 1, http://links.lww.com/SLA/E936).

### Postoperative Complications and Recovery

Overall postoperative complication rates were 42% and 23% after total and distal gastrectomy, respectively (Table 3). Complication severity was Clavien-Dindo grade I to II in 57% after total (n=65/115) and 53% after distal gastrectomy (n=55/104). Textbook outcome was achieved for 64% of patients after total and 74% after distal gastrectomy. Postoperative 30-day mortality after RAMIG was 1%.

Median hospital stay was 9 days (IQR: 7–14) after total gastrectomy (84% ERAS) and 9 days (IQR: 7–11) after distal RAMIG (61% ERAS). Hospital stay was shorter if ERAS guidelines were applied (n=472) compared with no ERAS [median 8 days (IQR: 7–10) vs. 10 days (IQR: 8–14)]. For ERAS patients with textbook outcome (n=359), median hospital stay was 8 days (IQR: 6–10) after total and 8 days (IQR: 7–9) after distal gastrectomy.

### Intraoperative Drain Placement

Surgical drains were often placed during total (80%) and distal gastrectomy (90%). Most centers (n=21) placed intraoperative drains as part of routine practice to detect and drain a potential leakage or for bleeding control, whereas 4 centers did not. These 21 centers routinely inserted a drain near the esophagojejunal/gastrojejunal anastomosis, and several centers (n=4) standardly placed a second drain near the duodenal stump or in the perihepatic region. Median hospital stay without routine perianastomotic drains was 3 days shorter than observed after standard intraoperative drain placement (Table 6). Without intraoperative drain insertion during total gastrectomy or with standard drain placement, comparable complication severity, and rates of complications (42% and 42%), anastomotic leakage (11% and 10%), reoperations (7% and 9%), and additional postoperative drain placement (18% and 16%) were observed. Distal gastrectomy showed similar results (Table 6).

**TABLE 6:** Perioperative Surgical Outcomes for Routine Drain Placement During RAMIG (n=758)

Routine intraoperative drain placement, n=758*	No drain, In total: n=103	1 or more drains, In total: n=633
Total gastrectomy (n=272)	**n=55 (100%)**	**n=217 (100%)**
Hospital stay, days, median [IQR]	7 [6–10]	10 [8–15]
Overall postoperative complications	23 (42)	91 (42)
Anastomotic leakage	6 (11)	21 (10)
Duodenal stump leakage	2 (4)	3 (1)
Chyle leakage	0	2 (1)
Most severe Clavien-Dindo grading		
Grade 0 (no complications)	32 (58)	125 (58)
Grade 1–3a	19 (35)	68 (31)
Grade ≥3b	4 (7)	24 (11)
Reoperation	4 (7)	20 (9)
Additional drain placement required	10 (18)	35 (16)
Distal gastrectomy (n=464)*	**n=48 (100%)**	**n=416 (100%)**
Hospital stay, days, median [IQR]	6 [4–8]	9 [7–11]
Overall postoperative complications	15 (31)	93 (22)
Anastomotic leakage	2 (4)	9 (2)
Duodenal stump leakage	1 (2)	3 (1)
Chyle leakage	1 (2)	1 (0.2)
Most severe Clavien-Dindo grading		
Grade 0 (no complications)	33 (70)	322 (78)
Grade 1–3a	9 (19)	62 (15)
Grade ≥ 3b	5 (11)	30 (7)
Reoperation	5 (11)	26 (6)†
Additional drain placement required	5 (10)	30 (7)

* There was 1 missing (0.1%) for intraoperative drain placement.

† One patient underwent a reoperation for removal of the drain tube, without having any other complications.

## DISCUSSION

This worldwide multicenter study presents an international cohort of currently applied RAMIG techniques with its associated perioperative surgical outcomes and short-term oncological findings. The observed perioperative outcomes demonstrated high surgical quality of RAMIG. Differences in RAMIG techniques among centers were identified predominantly for reconstruction and anastomotic techniques, extent of lymphadenectomy, omentectomy, ERAS application, and intraoperative drain placement.

The perioperative outcomes after RAMIG showed high quality of surgery. This is illustrated by our results after total and distal gastrectomy showing high lymph node yield (median 31 and 34 nodes), rate of ≥15 retrieved lymph nodes (92% and 96%) and radicality (93% and 96%), acceptable rates of overall postoperative complications (42% and 23%) and anastomotic leakage (10% and 2%), and low 30-day mortality (1%). Several multicenter randomized trials and population-based studies in gastric cancer surgery showed comparable nodal yield (median: 20–47 nodes), radicality (90%–100%), overall complications (15%–43%), anastomotic leakage (1%–9%), and postoperative mortality (0.4%–5%).^9–13,44–47^ Two previous American studies as well as 7 previous studies from China, Japan, and Korea (among which 3 randomized trials) showed similar good outcomes after RAMIG, all originating from high-volume centers.^23–26,31,48–51^ Furthermore, a previous retrospective study was conducted using the multicenter IMIGASTRIC registry after propensity score matching to compare outcomes after for open, laparoscopic, and robot-assisted gastrectomy.^30^ This registry-based research also reported similar surgical and oncological outcomes to our findings, although textbook outcome was not assessed. Importantly, higher textbook outcome rates were found for RAMIG after total (64%) and distal gastrectomy (74%) in the current study than the 22% to 55% textbook outcome after mostly laparoscopic and open gastrectomy that was reported in 4 population-based studies from different Western countries.^46,47,52,53^ Only one of these nationwide studies included robotic gastrectomies, showing 52% textbook outcome in the entire American population, or up to 60% when only including high-volume centers.^47^ The better results found in the present study could be explained by including experienced high-volume centers and surgeons in the UGIRA Gastric Registry, and is further supported by using the robotic approach for gastrectomy, which is also a factor that could reduce complications and hospital stay.^28,29,54–56^ Indeed, one previous study (high-volume, single center) found 73% textbook outcome after RAMIG.^32^ Although RAMIG is not yet applied on large scale internationally, these perioperative surgical and oncological outcomes are concordant with previous results from high-volume expert centers, set a quality standard for RAMIG, and can be used as international reference standard in gastric cancer surgery.

In general, most centers adhere to one particular anastomotic technique per gastrectomy type and then optimize their technique as much as possible to achieve their best outcomes, especially regarding anastomotic leakage rates. The observed anastomotic leakage rates varied per technique. Low leakage rates were found for linear stapled (6%) and hand-sewn (8%) anastomosis, whereas circular stapling frequently showed leakage (21%). This variation in leakage rates likely reflects a learning curve for circular stapling, and may be secondary due to differences in patient factors, disease stage, and surgical experience per center. The higher leakage rate after circular stapling might also result from the technique itself. A previous meta-analysis (n=2983) showed significantly more anastomotic leakage and complications after circular compared with linear stapling.^57^ Few studies were published on this topic, none including robotic procedures.^57–59^ Although firm conclusions based on the current study cannot be made as patients were not specifically matched and surgeon experience was not corrected for, our results certainly warrant further prospective studies to determine whether linear stapled and hand-sewn anastomoses may be superior to circular stapling.

Extent of lymphadenectomy during RAMIG was ≥D1+ (99%), resulting in high lymph node yield [34 nodes (IQR: 24–47)]. For cT1N0-stage gastric cancer, D1+ was performed most often (54%) followed by D2 (37%) and D2+ (10%). Although a D1+ for this patient subgroup corresponds to the 5th JGCA guidelines, multiple previous studies suggested that D2 lymphadenectomy may be necessary as well for cT1N0 tumors since stations 11d and 12a regularly showed nodal metastases, especially in Western patients.^41,60–64^ In the present study, advanced disease stages were predominantly treated with more extensive lymphadenectomy (D2/D2+ in 77%), adhering to the JGCA guidelines. In our RAMIG cohort, intraoperative bleeding and pancreatic/splenic injury during D2/D2+ rarely occurred, indicating that RAMIG is safe for performing extensive lymphadenectomy.

Although intraoperative frozen sections to secure the resection margin were not utilized for the majority of RAMIG procedures (74%), radicality was high for RAMIG after total (93%) and distal gastrectomy (96%), and concordant to previous nonrobotic trials with mainly advanced gastric cancer.^9–13,44–47^ Most irradical resections (63%) were diffuse type tumors, which are well known to result in positive resection margins more often.^44,65–68^

Although hospital stay was acceptable after total [9 days (IQR: 7–14)] and distal gastrectomy [9 days (IQR: 7–11)], ERAS principles were applied in only 84% and 61% of cases. Furthermore, routine intraoperative perianastomotic drain placement frequently occurred (86%). Previous studies showed that implementing ERAS accelerates recovery and reduces hospitalization after gastroesophageal cancer surgery without increasing complication rates.^43,69,70^ In this context, a previous meta-analysis demonstrated that refraining from routine perianastomotic drain placement reduced length of hospital stay.^71^ Wider adaptation of ERAS protocols could further improve outcomes after RAMIG.

Western patients had higher age, BMI, ASA classification, and comorbidities than Eastern patients, which is well known from literature.^72^ Furthermore, total gastrectomy was frequently performed, reflecting advanced disease stages, and proximal gastrectomy was mainly performed in the Asian population, as previously established.^72^ Future cross-continental studies with larger sample size should further evaluate intercontinental differences in RAMIG techniques and outcomes in-depth.

Since the participating centers registered all their RAMIG cases, also including the very first cases within their learning curve, our findings should be interpreted within this context. The MIG learning curve has been estimated at 20 to 95 cases depending on studied outcomes (ie, operating time, blood loss, complications, and lymphadenectomy), and may be shorter for RAMIG, especially for experienced laparoscopic surgeons.^18–22,73–75^ A shorter RAMIG proficiency gain curve probably underlies technical advantages of robotic surgery, including improved dexterity and magnified 3-dimensional visualization. The benefit of robot-assisted surgery is most evident for technical steps including the anastomosis and lymphadenectomy, and in challenging cases such as salvage surgery. Although our results already showed high surgical quality, including learning curve cases implies that the reported perioperative outcomes after RAMIG in the present study are not yet optimal and could be further improved.

This study has limitations. Although expert centers use RAMIG as standard approach for all gastrectomies, centers in the early phase of their learning curve may carefully select their first few patients for RAMIG. This might translate into lower risk of surgery and relatively good perioperative outcomes for this small subgroup of patients, but on the contrary might also translate into slightly higher risk of surgery by performing RAMIG during a surgeon’s learning curve. However, in order to present a realistic overview of the current stance of RAMIG, we consider it a strength to also retrieve data from centers in their RAMIG learning curve. Second, despite that all data were collected prospectively and uniform definitions (GCCG) were used, differences between centers could exist in reporting their complications, possibly introducing hospital reporting bias. Last, to guarantee anonymous data collection and facilitate patient privacy, the registry has limited follow-up, therefore impeding survival and quality of life analyses. Nonetheless, this study is based on an international population with prospective data from high-volume robotic centers, and is currently the largest published RAMIG cohort. Although not all known RAMIG centers contributed in this registry, the overview can be considered representative for worldwide practice of RAMIG. Furthermore, the UGIRA Gastric Registry facilitates international comparison as uniform definitions were used and stimulates standardization for gastric cancer surgery and RAMIG.

In conclusion, this worldwide multicenter study presents an overview of the currently applied surgical techniques with their respective perioperative outcomes after RAMIG. These findings from the UGIRA Gastric Registry demonstrated high surgical quality, set a quality standard for RAMIG and can be used as international reference standard. The optimal RAMIG techniques in terms of appropriate perioperative surgical outcomes and short-term oncological results should be further explored.

## Supplementary Material
