# Supplementary material for: Surgical Techniques and Related Perioperative Outcomes After Robot-assisted Minimally Invasive Gastrectomy (RAMIG): Results From the Prospective Multicenter International Ugira Gastric Registry
Source: Ann Surg. 2023 Nov 3;280(1):98–107. doi: 10.1097/SLA.0000000000006147 (PMC11161237; doi:10.1097/SLA.0000000000006147)
Supplement: Supplementary file 1 [file sla-280-098-s001.docx]

**Surgical techniques and related perioperative outcomes after robot-assisted minimally invasive gastrectomy (RAMIG): results from the prospective multicenter international UGIRA Gastric Registry.**

**AUTHORS**: Cas de Jongh^1^ MD, Fabio Cianchi^2^ MD PhD, Takahiro Kinoshita^3^ MD PhD, Feike Kingma^1^ MD PhD, Micaela Piccoli^4^ MD FACS, Attila Dubecz^5^ MD PhD, Ewout Kouwenhoven^6^ MD PhD, Marc van Det^6^ MD PhD, Tom Mala^7^ MD PhD, Andrea Coratti^8^ MD PhD, Paolo Ubiali^9^ MD PhD, Paul Turner^10^ MD PhD, Pursnani Kish^10^ MD PhD, Felice Borghi^11,12^ MD PhD, Arul Immanuel^13^ MD PhD, Magnus Nilsson^14^ MD PhD, Ioannis Rouvelas^14^ MD PhD, Jens Peter Hӧlzen^15^ MD PhD, Philippe Rouanet^16^ MD PhD, Olivier Saint-Marc^17^ MD PhD, David Dussart^17^ MD PhD, Alberto Patriti^18^ MD PhD, Francesca Bazzocchi^19^ MD PhD, Boudewijn van Etten^20^ MD PhD, Jan Willem Haveman^20^ MD PhD, Marco De Prizio^21^ MD PhD, Flávio Sabino^22^ MD PhD, Massimo Viola^23^ MD PhD, Felix Berlth^24^ MD PhD, Peter Philip Grimminger^24^ MD PhD, Franco Roviello^25^ MD PhD, Richard van Hillegersberg^1^ MD PhD, Jelle Ruurda^1^ MD PhD, UGIRA Collaborative Group.

**COLLABORATORS (UGIRA Collaborative Group):**

Giuseppe Barbato^2^, Tamae Takeuchi^3^, Masahiro Yura^3^, Francesca Pecchini^4^, Barbara Mullineris^4^, Melissa Kemeter^5^, Luca Giulini^5^, Dag Førland^7^, Michele Di Marino^8^, Angela Tribuzi^8^, Federica Maffeis^9^, Jaqueline Velkoski^9^, Ioannis Sarantitis^10^, Alessandra Marano^11^, Manuela Robella^12^, Helen Jaretzke^13^, Motonari Ri^14^, Mazen Juratli^15^, Anne Mourregot^16^, Filippo Petrelli^18^, Alessia Biancafarina^21^, Vittoria Barbieri^23^, Eren Uzun^24^, Luigi Marano^25^, Alessia d'Ignazio^25^, Luca Resca^25^.

**SUPPLEMENTARY MATERIAL**

1. ***Supplementary Methods.*** Participating centers. Page no. 1

- ***Supplementary Figure 1.*** A world map showing the location of all Page no. 1

25 participating centers.

1. ***Supplementary Figure 2***. Surgical techniques and anastomotic leakage Page no. 2

per continent.

1. ***Supplementary Table 1.*** Differences in patient characteristics for the Page no. 3

Western versus Eastern patients subgroups after RAMIG.

1. ***Supplementary Table 2*.** Perioperative outcomes for per type of Page no. 4

lymphadenectomy during RAMIG.

1. ***Supplementary Table 3.*** Radicality and frozen sections per type of Page no. 5

gastrectomy.

***Supplementary Methods.*** **Participating centers.**

The following 25 centers participated in this study (Supplementary Figure 1): University Medical Center Utrecht (The Netherlands), University Medical Center Careggi (Italy), National Cancer Center Hospital East (Japan), Civile Baggiovara Hospital Modena (Italy), Hospital ZGT Almelo (The Netherlands), Oslo University Medical Center (Norway), University Medical Center Siena (Italy), Newcastle upon Tyne Hospitals NHS (United Kingdom), Misericordia Hospital Grosseto (Italy), Hospital Santa Maria degli Angeli Pordenone (Italy), Lancashire Teaching Hospitals NHS (United Kingdom), General Hospital Cuneo (Italy), Candiolo Cancer Institute Torino (Italy), Karolinska University Medical Center Stockholm (Sweden), Montpellier Cancer Institute (France), University Medical Center Münster (Germany), Nürnberg Paracelsus University Medical Center (Germany), Hospital Régional Orléans (France), General Hospital Marche Nord (Italy), San Giovanni Rotondo Hospital IRCCS (Italy), University Medical Center Groningen (The Netherlands), General Hospital Arezzo (Italy), National Cancer Institute Rio de Janeiro (Brasil), General Hospital Tricase (Italy) and University Medical Center Mainz (Germany).

***Supplementary Figure 1.*** A world map showing the location of all 25 participating centers.

***
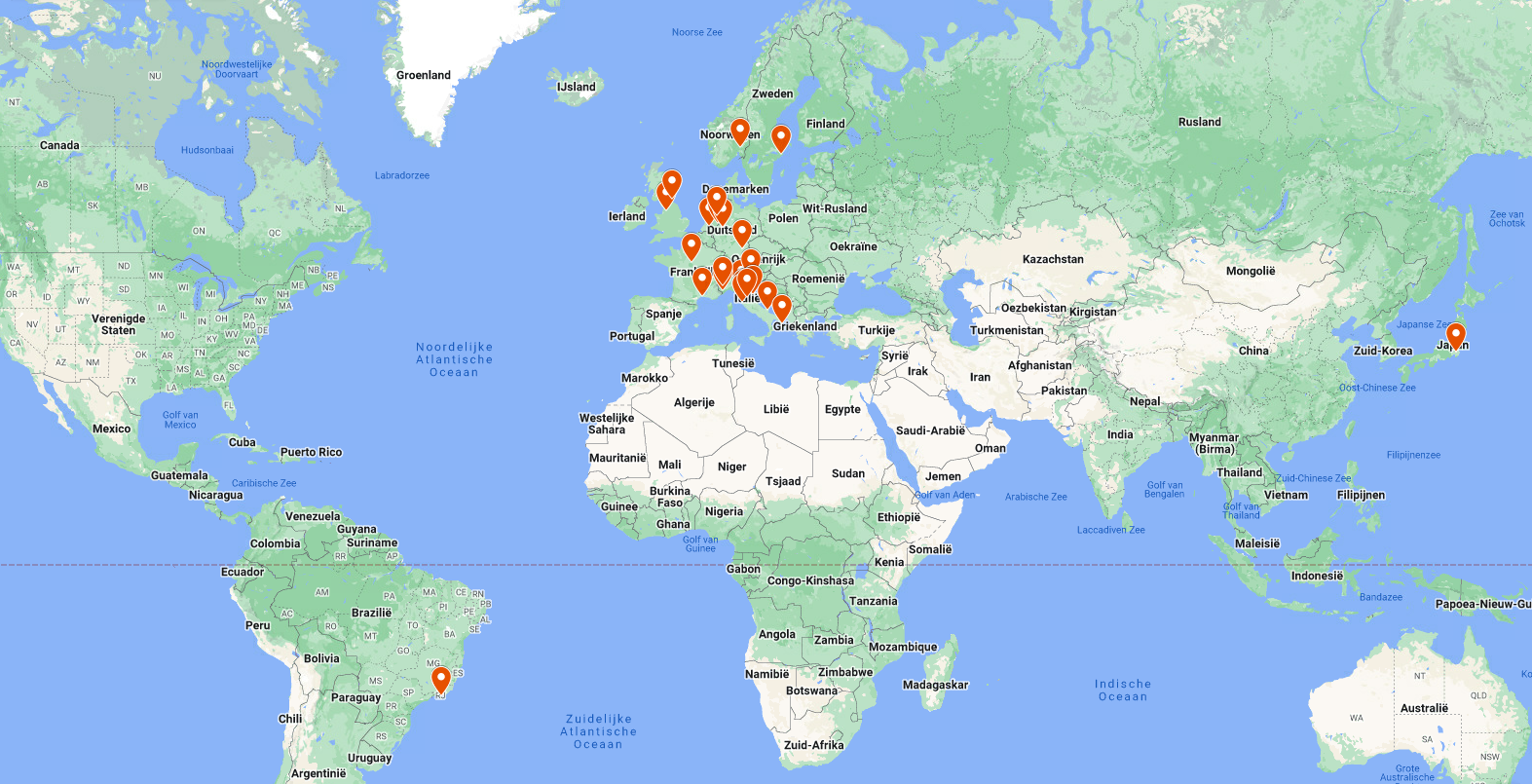
***

***Supplementary Figure 2.*** Surgical techniques and anastomotic leakage per continent in Europe (n=648), Asia (n=98) and South-America (n=11).


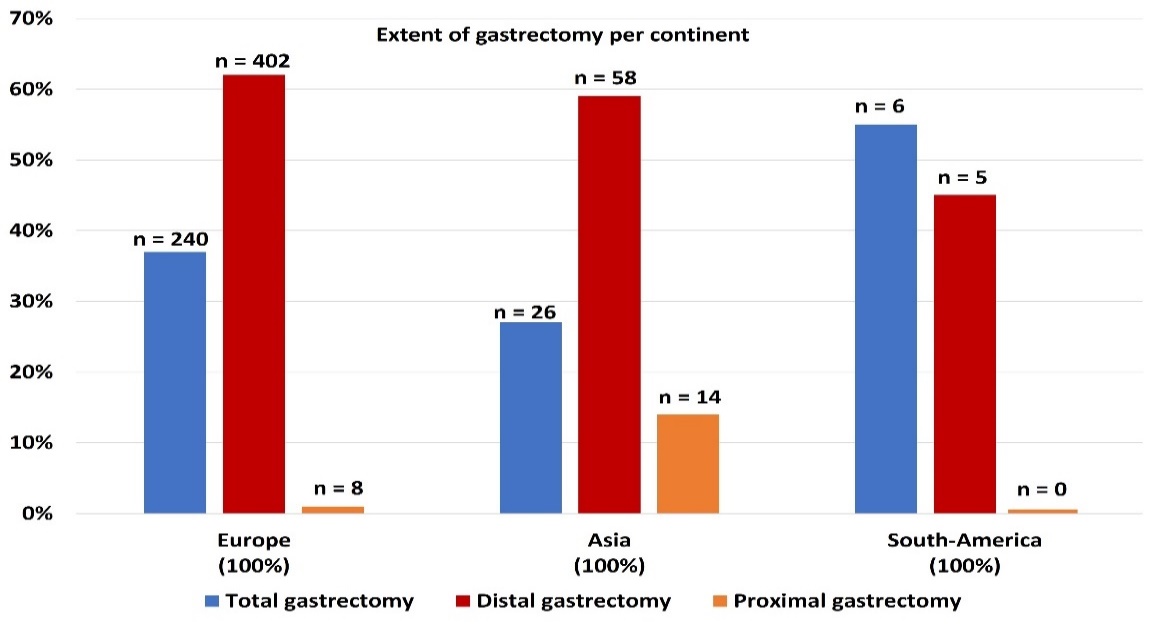


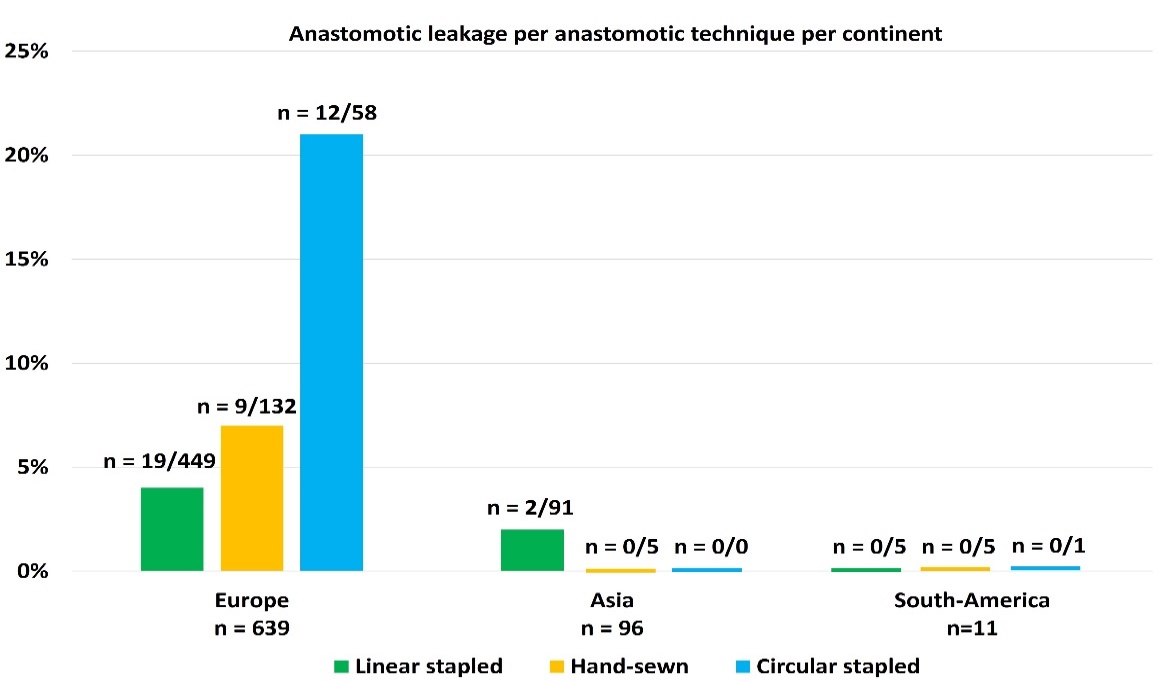


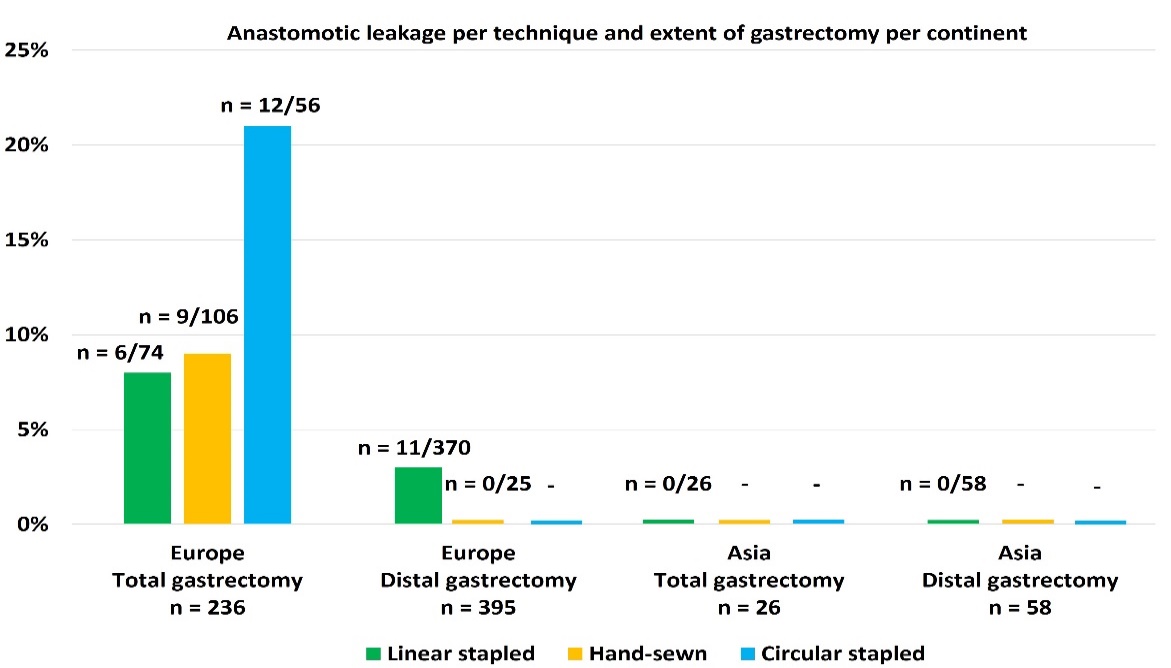


In the Asian cohort, anastomotic leakage developed in two patients from in total 14 proximal gastrectomies (14%).

The anastomotic leakage rates seemed lower in the Asian sub-cohort, but numbers are too small to draw any final conclusions.

| **Western and Eastern patients**  **n = 759** | **Western patients**  **n = 661 (100%)** | **Eastern patients**  **n =98 (100%)** |
| --- | --- | --- |
| **Age** *years* (median [range]) | 70 [61 – 77] | 69 [60 – 75] |
| **Sex**  Male  Female | 361 (56)  289 (44) | 60 (60)  38 (40) |
| **BMI** *kg/m^2^* **(**mean [SD]) | 25.2 ±4.5 | 22.8 ±3.5 |
| **ASA-classification**  1  2  3  4 | 40 (6)  358 (56)  231 (36)  7 (1) | 16 (16)  80 (82)  2 (2)  0 (0) |
| **Previous thoracic or intra-abdominal surgery (yes)** | 200 (31) | 29 (30) |
| **Any comorbidity** | 440 (69) | 56 (57) |
| **Pulmonary comorbidity** | 83 (13) | 6 (6) |
| **Cardiovascular comorbidity** | 298 (47) | 46 (47) |
| **Gastrointestinal comorbidity** | 64 (10) | 1 (1) |

***Supplementary Table 1.*** Differences in patient characteristics for the Western versus Eastern patients subgroups after RAMIG.

IQR = interquartile range. BMI = Body Mass Index (kg/m^2^). SD = standard deviation. ASA = American Society of Anesthesiologists. Percentages may differ from 100% due to rounding. For missing values, please see Table 1.

***Supplementary Table 2.*** Perioperative outcomes for per type of lymphadenectomy during RAMIG.

| **Extent of lymphadenectomy ***  **n = 756** ^a^ | **D1**  **n = 10 (100%)** | **D1+**  **n = 214 (100%)** | **D2**  **n = 443 (100%)** | **D2+**  **n = 89 (100%)** |
| --- | --- | --- | --- | --- |
| **Lymph node yield** *nodes* (median [IQR]) | 27 [21 – 35] | 33 [23 – 47] | 33 [23 – 45] | 40 [27 – 58] |
| **Operating time** *minutes* (median [IQR]) | 208 [178 – 364] | 294 [240 – 375] | 295 [237 – 358] | 315 [255 – 378] |
| **Blood loss** *mL* (median [IQR]) | 150 [100 – 300] | 100 [50 – 200] | 100 [50 – 200] | 100 [35 – 200] |
| **Conversion** | 0 (0) | 6 (3) | 25 (6) | 4 (4) |
| **Intraoperative bleeding**  **Intraoperative pancreatic injury** | 0 (0)  0 (0) | 1 (0.5)  0 (0) | 9 (2)  0 (0) | 0 (0)  0 (0) |
| **Intraoperative splenic injury** | 0 (0) | 1 (0.5) | 2 (0.5) | 1 (1) |

IQR = interquartile range. Bold indicates statistical significance.

* According to the 5^th^ definitions of the Japanese Gastric Cancer Association (JGCA) classification.

a. There were 3 missings (0.4%) for extent of lymphadenectomy. Furthermore, the clinical disease stage was insufficient to be stratified in the groups (cTxN0 or cNx) for 54 patients (7%).

***Supplementary Table 3.*** Radicality and frozen sections per type of gastrectomy.

| **Radicality**  **n = 745 RAMIG patients** ^a^ | **R0-resections**  **Negative resection margins** | **R1-resections ^b^**  **Positive resection margin(s)** |
| --- | --- | --- |
| **Intraoperative frozen section performed?**  Yes  No | 190 (96)  518 (95) | 7 (4)  30 (5) |
| **Only total gastrectomy patients (n=270)**  **Intraoperative frozen section performed?**  Yes  No | 87 (97)  164 (91) | 3 (3)  16 (9) |
| **Only distal gastrectomy patients (n=453) Intraoperative frozen section performed?**  Yes  No | 94 (97)  343 (96) | 3 (3)  13 (4) |
| **Only proximal gastrectomy patients (n=22) Intraoperative frozen section performed?**  Yes  No | 11 (92)  10 (90) | 1 (8)  1 (10) |

a. There were 14 missings (2%) for radicality.

b. Regarding all R1-resections (n=37), the Lauren histological subtypes were subdivided in diffuse type (n=19; 63%) or intestinal/mixed type (n=11; 37%). The remaining 7 patients (19%) had unknown Lauren subtype and were regarded as missings for the histological subtype.
